# Prevalence and impact of exploding head syndrome in a Japanese working population

**DOI:** 10.1093/sleep/zsaf007

**Published:** 2025-01-10

**Authors:** Uyanga Tsovoosed, Yukiyoshi Sumi, Yuji Ozeki, Akiko Harada, Hiroshi Kadotani

**Affiliations:** Department of Psychiatry, Shiga University of Medical Science, Otsu, Japan; Department of Communication Skills, Mongolian National University of Medical Sciences, Ulaanbaatar, Mongolia; Department of Psychiatry, Shiga University of Medical Science, Otsu, Japan; Department of Psychiatry, Shiga University of Medical Science, Otsu, Japan; Department of Medical Statistics, NCD Epidemiology Research Center, Shiga University of Medical Science, Otsu, Japan; Department of Psychiatry, Shiga University of Medical Science, Otsu, Japan

**Keywords:** parasomnias, exploding head syndrome, depression, anxiety, insomnia, quality of life assessment

## Abstract

**Study Objectives:**

Exploding head syndrome (EHS) is a parasomnia characterized by the perception of loud noises, or explosions inside the head during the sleep-to-wake transition. The prevalence of EHS remains unclear. This survey aimed to elucidate the prevalence of and factors associated with EHS in this cohort.

**Methods:**

As part of the Night in Japan Home Sleep Monitoring Study (NinjaSleep study), a cross-sectional survey was conducted among government employees in Koka City, Shiga Prefecture, Japan, in 2022. Participants were queried regarding their experiences with EHS as defined in the International Classification of Sleep Disorders, 3rd Edition, including sudden loud noises or sensations of explosions, subsequent abrupt awakenings and feelings of fright. Various standardized instruments were employed to evaluate depression, anxiety, insomnia, quality of life, and fatigue.

**Results:**

Of the 2081 employees invited to participate, 1878 completed the survey. After excluding respondents with epilepsy and incomplete responses, 1843 participants were deemed eligible for analysis. Among them, 46 (2.49%) reported experiencing sudden noises or sensations of explosions, with 23 (1.25%) meeting the diagnostic criteria for EHS. The EHS was significantly related to the scores on the Patient Health Questionnaire-9, Generalized Anxiety Disorder-7, Athens insomnia scale, and Chalder fatigue scale, even after adjusting for age, sex, body mass index, and categorized mean sleep duration.

**Conclusion:**

This study elucidates the prevalence of EHS among the Japanese population and underscores its potential association with insomnia symptoms and various psychological factors.

## Graphical Abstract

**Graphical Abstract:**
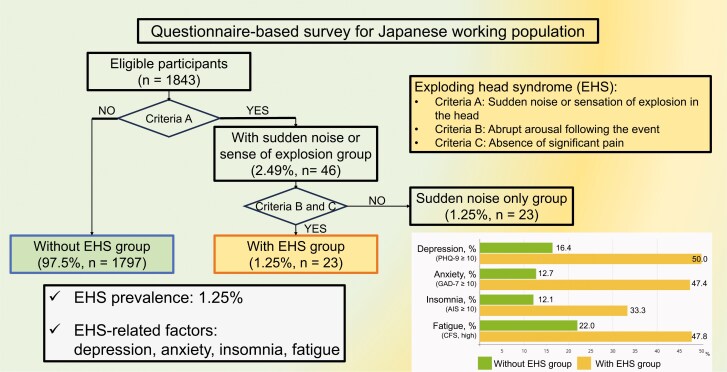

**Statement of Significance**

This study is the first to assess exploding head syndrome (EHS) prevalence in the Japanese population using ICSD-3 criteria, revealing significant associations between EHS and mental health factors, including depression, anxiety, insomnia, and fatigue. The findings highlight that EHS is linked to poorer mental quality of life, and these associations persisted even after adjusting for various demographic and sleep-related variables. Notably, our prevalence rates were lower than previously reported, suggesting that population characteristics and methodology, including stringent diagnostic criteria, may influence prevalence estimates. These results underscore the importance of accurate EHS assessment and the need for further longitudinal studies to explore the relationships between EHS, mental health, and well-being.

## Introduction

Exploding head syndrome (EHS) is a relatively understudied and poorly understood sleep disorder characterized by sudden auditory sensations, often described as explosions, bangs, or flashes of light, during the transition from wakefulness to sleep or during nocturnal awakenings. Despite its alarming nature, EHS is considered a benign condition [1, 2], typically devoid of pain or physical harm. However, the distressing nature of these episodes can significantly impact an individual’s quality of life (QOL) and overall well-being [3, 4].

While research on EHS has been limited, existing studies have reported varying prevalence rates across different populations [5]. The prevalence of EHS is not well understood especially in Asian countries. Furthermore, associations between EHS and other sleep-related disorders [6], such as insomnia, as well as psychological factors including anxiety and depression, have been suggested [7] but remain poorly understood.

Previous epidemiological studies on EHS have frequently targeted specific populations, such as individuals with a vested interest in sleep disorders [7], college students [8], and participants in sleep registries [9]. However, there is a need to establish the prevalence of EHS within the general population. Furthermore, many prior studies have defined EHS using a single item, typically a solitary question extracted from instruments like the Munich parasomnia screening measure (MUPS) [10]. The MUPS questionnaire, developed in 2007, is a self-rating measure used to assess various parasomnias. In the context of the MUPS, the EHS is typically defined as a single question that asks for the occurrence of a sudden, loud noise or sensation of an explosion in the head during sleep transitions or nocturnal awakenings. To address this limitation, it is imperative to differentiate EHS from conditions such as epilepsy and to employ robust criteria for diagnosing EHS, such as those outlined in the International Classification of Sleep Disorders, 3rd Edition (ICSD-3) [1].

Understanding the prevalence and correlation with EHS is essential for several reasons. First, it provides valuable insights into the burden of this relatively obscure sleep disorder, shedding light on its impact on daily functioning and QOL. Additionally, identifying the association between EHS and other sleep-related and psychological factors can inform targeted interventions and treatment approaches to alleviate symptoms and improve overall well-being.

We hypothesized that individuals with EHS would exhibit higher rates of insomnia and poorer measures of well-being than those without EHS. Furthermore, we anticipated significant associations between EHS and scores on measures of anxiety, depression, fatigue, and QOL. This study aimed to contribute to the understanding of EHS by investigating its prevalence and association with insomnia and measures of well-being, including anxiety symptoms, depression symptoms, fatigue, and QOL, within the Japanese working population. To the best of our knowledge, this study represents the first attempt to define EHS presence according to the ICSD-3 criteria in the working population.

## Methods

### Participants and procedures

A cross-sectional survey, both paper and web-based, was conducted as part of the Night in Japan Home Sleep Monitoring Study (NinJaSleep study). This survey focused on sleep and mental health within the Japanese working population [11, 12]. Specifically, questionnaire surveys were administered to local government employees in Koka City, a rural area in Shiga Prefecture, Japan [12]. All the employees (*n* = 2081) were asked to participate. A thorough review of patient histories, including epilepsy, was conducted.

The survey participants were government employees of Koka City who participated in the survey in 2022, which was conducted from November 1, 2022, to March 7, 2023. To assess comorbid otolaryngological and psychiatric disorders, participants were asked a general question regarding medical conditions they had been diagnosed with by a doctor: “Please tell us the name of the diseases you have been diagnosed with by a doctor?” Subsequently, participants were presented with a list of specific medical conditions, including “Sleep apnea syndrome,” “Insomnia,” “Depression,” and “Hypersomnia/narcolepsy.” Additionally, participants could specify any other conditions not listed. Participants were also asked to take medication to sleep easily (hypnotics), for anxiety (anxiolytics), for depression (antidepressants), or for epilepsy (antiepileptic drugs). This format allowed participants to identify relevant disorders without needing to interpret technical terms.

**Figure 1.**
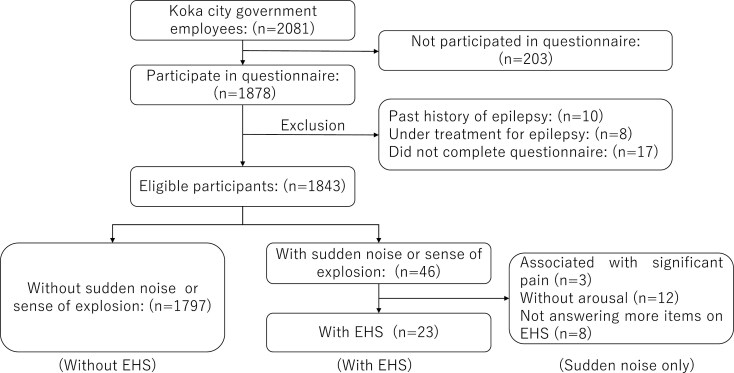
Flow diagram of the participant selection process. EHS: exploding head syndrome.

The exclusion criteria were as follows: participants with incomplete or invalid responses; individuals with a history of epilepsy or under epilepsy treatment because those had the potential for their aura symptoms to be mistaken for EHS. (Figure 1).

Approval for the study protocol (R2017–111) was obtained from the Ethics Committee of the Shiga University of Medical Science. The study was registered at UMIN-CTR (UMIN000028675, August 15, 2017) and ClinicalTrials.gov (NCT03276585, August 3, 2017). Informed consent was obtained from each participant prior to their participation. The datasets analyzed in this study can be made available by contacting the corresponding authors.

### Definition of EHS

EHS was assessed using three items derived from the ICSD-3 [1]. Criteria A–C stipulate that the individual must report: A) a sudden, loud noise or sensation of explosion in the head, typically at the wake–sleep transition or during nocturnal awakenings; B) an abrupt arousal following the event, often accompanied by feelings of fright; and C) the absence of significant pain associated with the experience [1].

In this study, participants were classified into three groups based on their fulfillment of specific criteria.

1. Participants who did not meet criterion A were defined as the “without EHS” group.
2. Participants who met criteria A, B, and C were defined as the “with EHS” group.
3. Participants who met only criterion A but not criteria B and C were defined as the “sudden noise only” group.

### Measures

The questionnaire included demographic variables, such as age, sex, body mass index (BMI), smoking status, and alcohol consumption. Participants’ mean sleep duration (mean sleep duration = (weekday sleep duration × 5 + weekend sleep duration × 2)/7) and the use of hypnotic medication, were also documented. We categorized mean sleep duration into three groups: short (<7 h), normal (7–8 h), and long (>8 h). This approach aligns with the literature indicating that both short and long sleep durations can be associated with health risks [13].

Participants were initially queried, “When dozing off or falling asleep, have you ever felt a sudden explosion in your head (e.g., bang, bang) or had the sensation that an explosion had occurred inside your skull?” Responses were categorized as either “yes” or “no.” Additionally, the participants were asked to report the frequency of sudden explosion sensations in their heads per month. Subsequently, participants who responded affirmatively were prompted by two additional inquiries. First, “Did you wake up suddenly afterwards?” Possible responses included “yes” and “no,” with an affirmative response indicative of EHS (criterion B of the ICSD-3 [1]). The final question pertained to pain experienced during the event, with response options including “no pain,” “little pain,” and “severe pain.” Participants reporting “no pain” or “little pain” were classified as exhibiting symptoms of EHS (criterion C of ICSD-3 [1]).

The Japanese version of Patient Health Questionnaire-9 [14] (PHQ-9) is a 9-item screening tool designed to assess depression severity in clinical and research settings [15]. Scores range from 0 to 27; higher scores indicate greater depression. Suspected and definite depression was defined by PHQ-9 scores ≥ 10. The PHQ-9 has a sensitivity and specificity of 0.85 (95% confidence interval (CI):0.79–0.89) and 0.85 (95% CI: 0.82–0.87) to detect major depression, respectively [16].

The Japanese version Generalized Anxiety Disorder-7 [17] (GAD-7) scale comprises seven items assessing anxiety symptoms over the past two weeks. Participants rated the frequency of seven anxiety-related symptoms over the past two weeks on a scale ranging from 0 (not at all) to 3 (nearly every day). Total scores range from 0 to 21, with higher scores indicating an increased severity of anxiety symptoms [18]. Scores ≥ 10 indicate suspected and definite anxiety.

Insomnia symptoms were evaluated using the Japanese version of the Athens insomnia scale (AIS) [19], a validated eight-item self-report questionnaire that assesses insomnia symptoms over the past month. The total score was calculated (range: 0–24), with lower scores indicating fewer insomnia symptoms. Subjects with AIS scores of ≥ 10 are expected to be diagnosed with insomnia [20] and moderate-to severe insomnia [21]. Thus, we classified AIS total scores of ≥10 as definite insomnia.

The severity of fatigue was measured using the Chalder fatigue scale (CFS) [22]. The reliability and validity of the Japanese version of the CFS for evaluating the severity of fatigue in students were previously confirmed [23] with higher scores indicating a greater degree of fatigue. Fatigue was defined as a score of ≥16 on the Japanese version of the CFS [23]. We used the median to separate high and low-fatigue subjects.

Health-related QOL was assessed using the SF-8, which consists of eight items and is divided into physical component summary (PCS) and mental component summary (MCS) scores [24]. Each item was converted to a 0–100 range based on Japanese population norms, with a score of 50 marking the national standard value [25]. Higher PCS and MCS scores indicate better health status, with scores above 50 considered above average and those below 50 considered below average for the Japanese population. This calculation approach does not involve additional weighting or coefficients for each subscale, consistent with SF-8 scoring guidelines specific to the Japanese population.

### Statistical analysis

We examined the association between “without EHS” and “with EHS” groups using the χ² test for categorical data and *t*-tests for continuous data. Subsequently, logistic regression analysis was used to identify variables independently associated with EHS. Several regression models were applied, including an unadjusted model and two subsequent models.

The unadjusted model predicted the EHS based on depressive syndrome (PHQ-9), anxiety syndrome (GAD-7), insomnia (AIS), and fatigue (CFS). Model 1 was controlled for the effects of age and sex. In Model 2, adjustments included age, sex, BMI, and categorized mean sleep duration.

Each variable was analyzed independently in separate logistic regression models because of their distinct hypotheses (Table S1).

Additionally, a correlation analysis was conducted using the PHQ-9, GAD-7, CFS, and AIS scores (Table S2). As an additional analysis, we compared the “with EHS” and “sudden noise only” groups (Table S3). To compare the frequency of explosive sound experiences between participants with and without EHS, a Mann–Whitney *U* test was performed. We conducted additional analysis as a part of sensitivity analysis comparing without EHS group to the experiencing sudden noise or sense of explosion group. Table S4 presents the characteristics of study participants in these groups. Additionally, we included a supplementary logistic regression analysis (Table S5) to assess associations within these groups. These analyses offer a clearer perspective on potential differences between participants with sudden noise experiences and those without.

All analyses were performed using SPSS software (version 25.0; IBM, Armonk, NY), with statistical significance set at *p* < 0.05.

Assuming a prevalence of EHS as 5% and that insomnia is 3 times more common among those with EHS (30% vs. 10%), we will need to study 25 case participants and 937.5 control participants (962.5 in total) to be able to reject the null hypothesis that the exposure rates for case and controls are equal with probability (power) 0.8. The type I error probability associated with this test of this null hypothesis is 0.05. We used an uncorrected chi-squared statistic to evaluate this null hypothesis.

The study method and results are reported following the Strengthening the Reporting of Observational Studies in Epidemiology (STROBE) Statement for cross-sectional studies.

## Results

There were 2081 employees initially recruited for the questionnaire survey, with 1878 employees participating in 2022. After screening, 17 participants with incomplete or invalid responses, 10 individuals with a history of epilepsy, and 8 undergoing epilepsy treatment were excluded. Consequently, 1843 participants were eligible for analysis. Among these, 1797 reported no sudden noise or a sense of explosion, 46 experienced sudden noise or a sense of explosion, and 23 exhibited symptoms of EHS with the full criteria mentioned above (A, B, and C) (Figure 1).

In our sample, 38.2% and 61.8% of participants were males and females, respectively (Table 1). Additionally, 14.1%, 9.8%, and 11.4% of participants exhibited depression (PHQ-9 ≥ 10), anxiety (GAD-7 ≥ 10), and insomnia (AIS ≥ 10), respectively. The age range of the participants was 20–-76 years, with a mean age of 45.8 years. Sex, as assessed using the chi-squared test, showed no significant association with EHS. Detailed descriptive statistics for all variables are presented in Table 1.

**Table 1.** Characteristics of study participants

Characteristic	Total	Without EHS	With EHS	*P*-value
	(*n* = 1820)	(*n* = 1797)	(*n* = 23)	
Age, mean (SD), year	45.8 (12.9)	45.8 (12.8)	44.8 (13.6)	.812
Sex (female, *n* (%))	1124 (61.8)	1108 (61.7)	16 (69.6)	.438
BMI, mean (SD), kg/m^2^	22.6 (3.82)	22.7 (2.98)	22.3 (3.01)	.287
Current smokers, *n* (%)	199 (10.9)	197 (11.0)	0 (8.70)	.729
Alcohol (habitual drinking), *n* (%)	775 (42.6)	763 (42.5)	12 (52.2)	.639
Mean sleep duration, mean (SD), h	6.53 (1.11)	6.54 (1.11)	6.13 (0.95)	.870
Categorized mean sleep duration, *n* (%)				
Short		1183 (65.8)	16 (69.9)	.440
Normal		495 (27.5)	7 (30.4)	
Long		119 (6.60)	0.00	
Use of hypnotic medication, *n* (%)	63 (3.50)	61 (3.40)	2 (8.70)	.167
History of undertreatment sleep apnea	65 (3.60)	63 (3.50)	2 (8.70)	.183
PHQ-9 score, mean (SD)	4.7 (4.61)	4.68 (4.52)	10.8 (6.99)	**<.001**
PHQ-9 ≥ 10, *n* (%)	256 (14.1)	245 (16.4)	11 (50.0)	**<.001**
GAD-7 score, mean (SD)	3.75 (4.16)	3.69 (4.08)	8.60 (7.12)	**<.001**
GAD-7 ≥ 10, *n* (%)	179 (9.8)	170 (12.7)	9 (47.4)	**<.001**
AIS score, mean (SD)	5.05 (3.64)	5.00 (3.60)	8.86 (4.39)	.395
AIS ≥ 10, *n* (%)	208 (11.4)	201 (12.1)	7 (33.3)	**<.001**
CFS score, mean (SD)	15.4 (8.35)	15.3 (8.29)	23.3 (9.77)	.159
CFS, high fatigue, *n* (%)	406 (22.3)	395 (22.0)	11 (47.8)	**.003**
SF-8 MCS score, mean (SD)	46.9 (7.98)	47.0 (7.92)	41.6 (10.45)	**.020**
SF-8 MCS score > 50, *n* (%)	764 (42.0)	759 (42.2)	5 (21.7)	**.048**
SF-8 PCS score, mean (SD)	47.6 (7.42)	47.7 (7.38)	43.0 (8.84)	.111
SF-8 PCS score > 50, *n* (%)	797 (43.8)	791 (44.0)	6 (26.1)	.085

EHS: exploding head syndrome; SD: standard deviation; BMI: body mass index; Short, normal, and long categorized mean sleep duration: <7, 7–8, and >8 h, respectively; PHQ-9: Patient Health questionnaire-9; GAD-7: 7-item Generalized Anxiety Disorder Scale; AIS: Athens insomnia scale; CFS: Chalder fatigue scale; CFS, high fatigue: CFS > median (22); SF-8: 8-item questionnaire for quality of life; MCS: mental component summary; PCS: physical component summary. *P*-values < 0.05 were indicated in bold.

**Figure 2.**
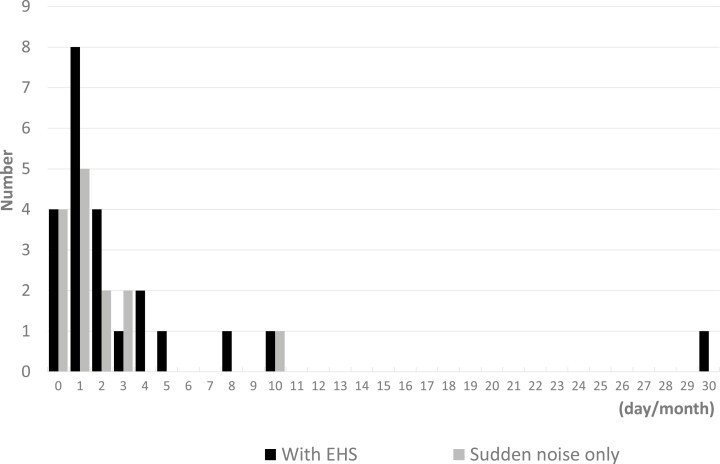
Histogram shows the frequency of sudden noise or sense of explosion. EHS: exploding head syndrome.

In both the EHS and sudden noise-only groups, the frequency of explosive sound experiences was nearly equivalent (Table S3). A total of 46 participants reported experiencing explosive sounds; however, only 38 participants provided responses to the current frequency question. This suggests that the remaining participants did not experience explosive sounds within the past month (Figure 2). Apart from these observations, no other discernible differences were noted between the EHS and sudden noise groups. Two (4.3 %) of the excluded individuals with a history of epilepsy or under epilepsy treatment had experienced sudden noise or a sense of explosion.

The PHQ-9, GAD-7, AIS, CFS, and QOL mental health scores differed significantly between participants with and without EHS. Further exploration using logistic regression analysis (Table 2) indicated that depressive symptoms, anxiety, insomnia, and fatigue were significantly associated with EHS in the unadjusted model. This association persisted in Models 1 and 2. The Mann–Whitney *U* test results indicated no significant difference in the frequency of explosive sounds between groups (Mann–Whitney *U* = 78.000, *Z* = −0.492, *p* = 0.623), suggesting that the presence of EHS does not significantly influence the frequency of these experiences. As a sensitivity analysis, we compared between without EHS group and with sudden noise or sense of explosion group, and the results were similar (**Table S4****and****S5**).

**Table 2.** Logistic regression analysis of factors associated with EHS

	Unadjusted		Model 1		Model 2	
	OR (95%CI)	ρ-value	OR (95% CI)	ρ-value	OR (95% CI)	ρ-value
PHQ-9 score	1.188 (1.120 to 1.260)	**<.001**	1.190 (1.121 to 1.263)	**<.001**	1.197 (1.125 to 1.274)	**<.001**
GAD-7 score	1.189 (1.112 to 1.270)	**<.001**	1.193 (1.115 to 1.276)	**<.001**	1.193 (1.114 to 1.277)	**<.001**
AIS score	1.233 (1.132 to 1.343)	**<.001**	1.236 (1.134 to 1.348)	**<.001**	1.241 (1.136 to 1.355)	**<.001**
CFS score	1.110 (1.059 to 1.163)	**<.001**	1.110 (1.059 to 1.164)	**<.001**	1.112 (1.060 to 1.167)	**<.001**

Model 1: Adjusted: age, sex.

Model 2: Adjusted: age, sex, BMI, categorized mean sleep duration (<7, 7–8, and >8 h).

EHS: exploding head syndrome; PHQ-9 score: scores of Patient Health Questionnaire-9; GAD-7 score: scores of 7-item generalized anxiety disorder scale; AIS score: scores of Athens insomnia scale; CFS score: scores of Chalder fatigue scale; OR: odds ratio; CI: confidence interval.

Additionally, our survey revealed a significant positive correlation among the measures employed in our study: the PHQ-9, GAD-7, AIS, and CFS (Table S2). There was no difference in the characteristics between participants with EHS and those with sudden noise in Table S3.

## Discussion

In this study, we investigated the prevalence of EHS and its association with insomnia and various measures of well-being, including anxiety, depression, fatigue, and QOL, in the Japanese population. Notably, this study is the first to define EHS presence according to the ICSD-3 criteria. Our statistical analysis revealed significant associations between EHS and PHQ-9, GAD-7, AIS, and CFS scores, even after adjusting for demographic and sleep-­related variable.

Notably, the prevalence rates observed in our study were lower than those reported previously. According to an online survey from the Netherlands in a study involving participants from a national sleep registry, the prevalence of EHS ranged from 2.6% (35 out of 1333) among participants without insomnia to 6.8% (60 out of 877) among participants with insomnia [9]. Fulda and colleagues reported a prevalence of 11.1% [10] utilizing a single item of the MUPS. Subsequent studies using the same measure have reported varying lifetime prevalence rates: 37.29% [7] in a UK study, 29.59% [7] in an international sample, 20% [8] in an Irish sample, and 18% [26] in a US sample. This discrepancy suggests that the characteristics of the populations included in prevalence studies significantly influenced the reported prevalence rates of EHS. The potential overestimation observed in previous studies may be attributed, at least in part, to the limitations of using a single question item from the MUPS. Despite this limitation, the estimated prevalence in our study remained notably lower than those reported in previous studies. Furthermore, the recruitment of participants from online forums dedicated to discussions of sleep paralysis and lucid dreaming may have resulted in experiences resembling EHS [1]. Higher prevalence due to participant bias has been reported when recruiting via the web [27]. Additionally, the possibility of inadequate exclusion criteria for epilepsy [28] or migraine-related [29] experiences among participants may have further contributed to this bias. Conversely, it is plausible that our study underestimated the prevalence of EHS because of the participants’ potential lack of familiarity with the condition, which may have hindered their ability to report relevant experiences accurately in the survey. Nevertheless, with a high participation rate of 88.5%, our prevalence estimate provided a representative snapshot of the prevalence of EHS in the target population.

In our logistic regression models, insomnia, anxiety, depression, and fatigue were significantly and independently associated with EHS. This aligns with previous findings that have shown associations between EHS and higher anxiety scores [7, 8]. Larger survey studies have demonstrated that insomnia and poor sleep quality [6] are important correlates of EHS and well-being [6–9]. Despite a previous study suggesting a higher prevalence of EHS in females [6] and significant differences in the age of individuals reporting EHS [6], and in line with findings from other studies, our investigation did not identify any sex or age differences.

Depression, anxiety, insomnia, fatigue, and QOL may be correlated and interactive, suggesting a spectrum of symptomatology. Further studies are required to explore these relationships in detail.

When adjusting for age, sex, BMI, and categorized mean sleep duration in Model 2 of the logistic regression analysis, EHS was found to be significantly associated with depression, anxiety, insomnia, and fatigue scores. These findings do not establish a definitive relationship between mental health problems and the occurrence of EHS as a state or trait. Further longitudinal studies are warranted to follow up on these findings and elucidate the nature of the relationship between EHS and mental health outcomes.

Regarding the limitations of our study, it is important to acknowledge that our assessments relied on self-ratings rather than face-to-face interviews, which could have affected the diagnoses of depression, anxiety, insomnia, fatigue, or other mental disorders. Therefore, the observed prevalence of EHS in our study may not fully reflect the true prevalence of this condition. Additionally, our sample consisted of a working population in a rural city in Japan, which limits the generalizability of our results to the broader Japanese population or other countries without further investigation. One limitation of our study is the lack of a defined time frame for assessing the frequency of sudden exploding sounds. This ambiguity may have led participants to report on non-current experiences, thereby introducing potential recall bias. Implementing a clearer time frame could help distinguish between recent and past episodes, offering a more accurate understanding of the relationship between current episodes of sudden exploding sounds and associated health outcomes. The role of fright during EHS episodes presents an intriguing dimension of this condition. Although fright was not a required criterion in this study, it remains unclear whether those who experience an arousal with fright during EHS episodes differ in meaningful ways from those who do not. Additionally, the finding that some participants reported hearing sudden noises without any arousal response may suggest that these experiences are more akin to dream recall. Exploring these nuances further could help clarify the full spectrum of EHS experiences and associated characteristics.

Another limitation of our study stems from its cross-sectional design, which precludes the determination of causality between EHS and depression, anxiety, and insomnia. While our analysis did not identify significant differences between the sudden noise-only group and the EHS group, it is important to note that the small sample sizes in these groups may have limited our ability to detect subtle differences, particularly in measures such as GAD-7 scores. Longitudinal studies can provide valuable insights into the changes in EHS symptoms, both transient and enduring, over time.

Therefore, further longitudinal studies with larger and more representative samples and rigorous follow-up procedures are essential to gain a more precise understanding of the EHS trajectory over time. It is also crucial to investigate the relationship between psychiatric symptoms and EHS.

A promising direction for future research would be to conduct multi-night polysomnography (PSG) studies or EEG recordings, potentially utilizing new wearable technologies. Such studies could offer detailed data on arousals, quantitative EEG (qEEG) measures, and other neurophysiological markers, helping to elucidate the underlying mechanisms of EHS. These methods could significantly improve our understanding of EHS and lead to more precise diagnostic criteria and targeted interventions. Given the variation in presentation and experience of EHS across studies, a consensus definition is needed to ensure consistency in measurement and reporting. Standardizing the definition of EHS would allow future epidemiological studies to employ a uniform criterion, which could facilitate more accurate and comparable data across diverse populations. This would ultimately improve our understanding of EHS prevalence, associated factors, and its impact on health outcomes.

In conclusion, this study represents the largest published sample to date on the examination of EHS, with a relatively high participation rate. Our findings suggest that the mental health components of QOL may be associated with the presence of EHS and that EHS is linked to depression, anxiety, insomnia, and fatigue scores. Moving forward, there is a compelling need to expand research on EHS, particularly through better prevalence studies that utilize appropriate criteria and standardized assessment protocols.

## Supplementary material

Supplementary material is available at *SLEEP* online.

zsaf007_suppl_Supplementary_Tables

## Data Availability

The data underlying this article will be shared on reasonable request to the corresponding author.
