# Supplementary material for: Prevalence and impact of exploding head syndrome in a Japanese working population
Source: Sleep. 2025 Jan 10;48(5):zsaf007. doi: 10.1093/sleep/zsaf007 (PMC12068057; doi:10.1093/sleep/zsaf007)
Supplement: zsaf007_suppl_Supplementary_Tables [file zsaf007_suppl_supplementary_tables.docx]

**SUPPLEMENTARY TABLES**

**Title: Prevalence and impact of Exploding Head Syndrome in a Japanese working population**

**Authors:** Uyanga Tsovoosed^1,2^, Yukiyoshi Sumi^1^, Yuji Ozeki^1^, Akiko Harada^3^, Hiroshi Kadotani^1^, and the NinJa Sleep Study Group

**Affiliation information for all authors:** ^1^Department of Psychiatry, Shiga University of Medical Science, Japan; ^2^Department of Communication Skills, Mongolian National University of Medical Sciences, Mongolia, ^3^Department of Medical Statistics, NCD Epidemiology Research Center, Shiga University of Medical Science

***Corresponding authors information:** Hiroshi Kadotani. Department of Psychiatry, Shiga University of Medical Science, 520-2192, Seta Tsukinowa-cho, Otsu, Shiga, Japan. Telephone: +81-77-548-2291. Fax: +81-77-543-9698

E-mail: kadotanisleep@gmail.com

**SUPPLEMENTARY TABLES**

**Table S1. Independent logistic regression models for each variable analyzed with EHS as the dependent variable**

|  | Unadjusted | | |
| --- | --- | --- | --- |
|  | OR | (95% CI) | ρ-value |
| Age | 0.994 | (0.963 to 1.026) | 0.708 |
| Sex | 1.421 | (0.582 to 3.472) | 0.440 |
| BMI | 1.009 | (0.908 to 1.121) | 0.869 |
| Smoking | 1.293 | (0.301 to 5.555) | 0.730 |
| Alcohol | 1.051 | (0.902 to 1.225) | 0.520 |
| Mean sleep duration | 0.761 | (0.562 to 1.029) | 0.076 |
| Use of hypnotic medication | 0.369 | (0.085 to 1.609) | 0.185 |
| PHQ-9 score | 1.188 | (1.120 to 1.260) | **<0.001** |
| GAD-7 score | 1.189 | (1.112 to 1.270) | **<0.001** |
| AIS score | 1.233 | (1.132 to 1.343) | **<0.001** |
| CFS score | 1.110 | (1.059 to 1.163) | **<0.001** |
| SF-8 higher mental health | 0.380 | (0.140 to 1.028) | 0.057 |
| SF-8 higher physical health | 0.449 | (0.176 to 1.144) | 0.093 |
| BMI, Body mass index; PHQ-9 score, scores of Patient Health Questionnaire-9; GAD-7 score, scores of 7-item Generalized Anxiety Disorder scale; AIS score, scores of Athens Insomnia Scale; CFS score, scores of Chalder Fatigue Scale; SF-8, 8 item questionnaire for quality of life; SF-8 Mental Component Summary score > 50 and Physical Component Summary score > 50 suggest higher mental and physical health compared to the Japanese national norms, respectively. P-values < 0.05 were indicated in bold. | | | |

**Table S2. Correlation table among the four questionnaires**

|  | | | | |
| --- | --- | --- | --- | --- |
|  | PHQ-9 score | GAD-7 score | CFS score | AIS score |
| PHQ-9 score | 1 | 0.799** | 0.705** | 0.729** |
| GAD-7 score | 0.799** | 1 | 0.622** | 0.661** |
| AIS score | 0.705** | 0.622** | 1 | 0.664** |
| CFS score | 0.729** | 0.661** | 0.664** | 1 |
| PHQ-9 score, scores of Patient Health Questionnaire-9; GAD-7 score, scores of 7-item Generalized Anxiety Disorder scale; AIS score, scores of Athens Insomnia Scale; CFS score, scores of Chalder Fatigue Scale. | | | | |

**Table S3. Comparison demographic table “with EHS” and “sudden noise only” groups**

|  | | | |  |
| --- | --- | --- | --- | --- |
|  | **Total** | **Sudden noise only** | **With EHS** | **ρ-value** |
|  | **(n= 46)** | **(n= 23)** | **(n= 23)** |  |
| Age, mean (SD), year | 47.0 (13.7) | 49.1 (13.8) | 44.8 (13.6) | 0.936 |
| Sex (female n, (%)) | 29 (63.0) | 13 (56.5) | 16 (69.6) | 0.359 |
| BMI, mean (SD), kg/m2 | 22.0 (3.04) | 21.2 (2.96) | 22.7 (2.98) | 0.944 |
| Current smokers n, (%) | 5 (10.9) | 3 (13.0) | 2 (8.70) | 0.636 |
| Alcohol (habitual drinking) n, (%) | 20 (43.4) | 8 (34.8) | 12 (52.2) | 0.349 |
| Mean sleep duration, mean (SD), hours | 6.17 (1.10) | 6.21 (1.26) | 6.13 (0.95) | 0.409 |
| Categorized mean sleep duration short n, (%) |  | 6 (26.1) | 7 (30.4) | 0.743 |
| normal |  | 0 (0.00) | 0 (0.00) |  |
| long |  | 17 (73.9) | 16 (69.6) |  |
| Use of hypnotic medication n, (%) | 5 (10.9) | 3 (13.0) | 2 (8.70) | 0.636 |
| History of undertreatment sleep apnea n, (%) | 2 (4.30) | 0 (0.00) | 2 (8.70) | 0.148 |
| FES/per month, median (IQR) | 1 (1–3) | 0 (0-2) | 1 (1-4) | 0.539 |
| PHQ-9 score, mean (SD) | 9.09 (7.02) | 7.35 (6.76) | 10.83 (6.99) | 0.858 |
| PHQ-9 ≥ 10 n, (%) | 20 (43.5) | 9 (50.0) | 11 (50.0) | 1.000 |
| GAD-7 score, mean (SD) | 7.00 (6.69) | 5.39 (5.96) | 8.60 (7.12) | 0.147 |
| GAD-7 ≥ 10 n, (%) | 14 (30.4) | 5 (31.3) | 9 (47.4) | 0.332 |
| AIS score, mean (SD) | 7.34 (4.92) | 5.82 (5.05) | 8.86 (4.39) | 0.469 |
| AIS ≥ 10 n, (%) | 13 (28.3) | 6 (30.4) | 7 (33.3) | 0.819 |
| CFS score, mean (SD) | 20.1 (10.58) | 16.9 (10.57) | 23.3 (9.77) | 0.887 |
| CFS, high fatigue n, (%) | 18 (39.1) | 7 (30.4) | 11 (47.8) | 0.227 |
| SF-8 MCS score, mean (SD) | 43.5 (9.77) | 45.3 (8.88) | 41.6 (10.45) | 0.436 |
| SF-8 higher mental health n, (%) | 14 (30.4) | 9 (39.1) | 5 (21.7) | 0.200 |
| SF-8 PCS score, mean (SD) | 44.1 (8.61) | 45.2 (8.44) | 43.0 (8.84) | 0.391 |
| SF-8 higher physical health n, (%) | 12 (26.1) | 6 (26.1) | 6 (26.1) | 1.000 |
| EHS, exploding head syndrome; SD, standard deviation; BMI, Body mass index; Short, normal, and long Categorized mean sleep duration: <7, 7-8, and >8 hours, respectively; FES, Frequency of Explosive Sound/per month; IQR, Interquartile Range; PHQ-9, Patient Health Questionnaire-9; GAD-7, 7-item Generalized Anxiety Disorder scale; AIS, Athens Insomnia Scale; CFS, Chalder Fatigue Scale; CFS, high fatigue, CFS>median (22); SF-8, 8-item questionnaire for quality of life; MCS, Mental Component Summary; PCS, Physical Component Summary. SF-8 Mental Component Summary score > 50 and Physical Component Summary score > 50 suggest higher mental and physical health compared to the Japanese national norms, respectively. | | | | |

**Table S4. Characteristics of study participants “without EHS” and “with sudden noise or sense of explosion”**

|  |  |  |  |  |
| --- | --- | --- | --- | --- |
| **Characteristic** | **Total** | **Without EHS** | **With sudden noise or sense of explosion** | **ρ-value** |
|  | **(n= 1843)** | **(n= 1797)** | **(n= 46)** |  |
| Age, mean (SD), year | 45.9 (12.9) | 45.8 (12.8) | 47.0 (13.7) | 0.497 |
| Sex (female n, (%)) | 1137 (61.7) | 1108 (61.7) | 29 (63.0) | 0.849 |
| BMI, mean (SD), kg/m2 | 22.6 (3.80) | 22.6 (3.82) | 22.0 (3.04) | 0.266 |
| Current smokers n, (%) | 202 (11.0) | 197 (11.0) | 5 (10.9) | 0.984 |
| Alcohol (habitual drinking) n, (%) | 783 (42.5) | 763 (42.4) | 20 (43.5) | 0.383 |
| Mean sleep duration, mean (SD), hours | 6.53 (1.11) | 6.54 (1.11) | 6.17 (1.10) | 0.348 |
| Categorized mean sleep duration short n, (%) |  | 1183 (65.8) | 33 (71.7) | 0.193 |
| normal |  | 495 (27.5) | 13 (28.3) |  |
| long |  | 119 (6.60) | 0.00 |  |
| Use of hypnotic medication n, (%) | 66 (3.60) | 61 (3.40) | 5 (10.9) | 0.007 |
| History of undertreatment sleep apnea | 65 (3.50) | 63 (3.50) | 2 (4.30) | 0.760 |
| PHQ-9 score, mean (SD) | 4.79 (4.65) | 4.68 (4.52) | 9.09 (7.02) | **<.001** |
| PHQ-9 ≥ 10 n, (%) | 265 (14.4) | 245 (16.4) | 20 (50.0) | **<.001** |
| GAD-7 score, mean (SD) | 3.77 (4.19) | 3.69 (4.08) | 7.00 (6.69) | **<.001** |
| GAD-7 ≥ 10 n, (%) | 184 (10.0) | 170 (12.7) | 14 (40.0) | **<.001** |
| AIS score, mean (SD) | 5.06 (3.66) | 5.00 (3.60) | 7.34 (4.92) | **0.002** |
| AIS ≥ 10 n, (%) | 214 (11.6) | 201 (12.1) | 13 (31.7) | **<.001** |
| CFS score, mean (SD) | 15.4 (8.38) | 15.3 (8.29) | 20.1 (10.58) | **0.015** |
| CFS, high fatigue n, (%) | 413 (22.4) | 395 (22.0) | 18 (39.1) | **0.006** |
| SF-8 MCS score, mean (SD) | 49.9 (7.99) | 47.0 (7.92) | 43.5 (9.77) | **0.009** |
| SF-8 higher mental health n, (%) | 773 (41.9) | 759 (42.2) | 14 (30.4) | 0.109 |
| SF-8 PCS score, mean (SD) | 47.5 (7.43) | 47.6 (7.38) | 44.1 (8.61) | 0.144 |
| SF-8 higher physical health n, (%) | 803 (43.6) | 791 (44.0) | 12 (26.1) | **0.015** |
| EHS, exploding head syndrome; SD, standard deviation; BMI, Body mass index; Short, normal, and long Categorized mean sleep duration: <7, 7-8, and >8 hours, respectively; PHQ-9, Patient Health Questionnaire-9; GAD-7, 7-item Generalized Anxiety Disorder scale; AIS, Athens Insomnia Scale; CFS, Chalder Fatigue Scale; CFS, high fatigue, CFS>median (22); SF-8, 8-item questionnaire for quality of life; MCS, Mental Component Summary; PCS, Physical Component Summary. SF-8 Mental Component Summary score > 50 and Physical Component Summary score > 50 suggest higher mental and physical health compared to the Japanese national norms, respectively. P-values < 0.05 were indicated in bold. | | | | |

**Table S5. Logistic regression analysis of factors associated with the “sudden noise or sense of explosion” compared to the “without EHS”**

|  | | | | | | |
| --- | --- | --- | --- | --- | --- | --- |
|  | **Unadjusted** |  | **Model 1** |  | **Model 2** |  |
|  | **OR (95%CI)** | **ρ-value** | **OR (95% CI)** | **ρ-value** | **OR (95% CI)** | **ρ-value** |
| PHQ-9 score | 1.146 (1.096 to 1.199) | **<0.001** | 1.153 (1.101 to 1.206) | **<0.001** | 1.162 (1.107 to 1.218) | **<0.001** |
| GAD-7 score | 1.138 (1.082 to 1.197) | **<0.001** | 1.146 (1.088 to 1.207) | **<0.001** | 1.148 (1.089 to 1.210) | **<0.001** |
| AIS score | 1.150 (1.077 to 1.228) | **<0.001** | 1.154 (1.081 to 1.232) | **<0.001** | 1.160 (1.084 to 1.241) | **<0.001** |
| CFS score | 1.066 (1.031 to 1.102) | **<0.001** | 1.068 (1.033 to 1.105) | **<0.001** | 1.071 (1.035 to 1.109) | **<0.001** |
| Model 1: Adjusted: Age, Sex | | | | | | |
| Model 2: Adjusted: Age, Sex, BMI, Categorized mean sleep duration (<7, 7-8, and >8 hours)  EHS, exploding head syndrome; PHQ-9 score, scores of Patient Health Questionnaire-9; GAD-7 score, scores of 7-item Generalized Anxiety Disorder scale; AIS score, scores of Athens Insomnia Scale; CFS score, scores of Chalder Fatigue Scale; OR, odds ration; CI: confidence interval. | | | | | | |
